# The Journey Toward Health Care Equity: Accredited Hospitals’ Alignment with Joint Commission Health Care Equity Standards Preimplementation

**DOI:** 10.1089/heq.2024.0036

**Published:** 2024-10-23

**Authors:** Jamie Patrianakos, Scott C. Williams, Brian Johnson, David W. Baker

**Affiliations:** ^1^ Department of Research, The Joint Commission, Oakbrook Terrace, Illinois, USA.; ^2^ Department of Business Development, The Joint Commission, Oakbrook Terrace, Illinois, USA.; ^3^ Healthcare Quality Evaluation and Improvement, The Joint Commission, Oakbrook Terrace, Illinois, USA.

**Keywords:** health equity, health-related social needs, SDOH, health disparities, Joint Commission, health care equity

## Abstract

**Background:**

The Joint Commission (TJC) released new health care equity (HCE) standards for hospitals with the goal of helping organizations monitor and improve equity of care. This study assessed the state of the field immediately following the release of the new standards to gain a baseline understanding of the field’s progress toward HCE.

**Methods:**

This was a cross-sectional observational study. An online questionnaire assessed four domains related to organization’s progress toward HCE, as it aligns with TJC’s accreditation and certification requirements: 1) leadership, 2) collaboration, 3) collecting and using data, and 4) provision of care. The questionnaire was distributed between April and June 2023. Included hospitals received an accreditation visit in 2022 or were scheduled for a visit after October 2024, leaving 2,098 eligible hospitals. A representative sample of 1,625 hospitals received the questionnaire.

**Results:**

In total, 340 hospitals (20.9%) responded to the questionnaire. Hospitals were mostly meeting the mark in the leadership domain, but many have addressed the other domains to a limited degree. Not-for profit hospitals and those that are part of a system were more likely to have made strides toward achieving compliance with HCE requirements, whereas behavioral health/psychiatric hospitals have made less progress in this area.

**Health Equity Implications:**

Many of the hospitals surveyed have made advances in the HCE-related topics covered in this study, but achieving HCE is only part of the journey toward overall health equity. Societal institutions must work together to address the social determinants of health for entire communities in addition to individual patient needs.

## Introduction

Factors outside of an individual’s control (e.g., social, economic, and policy) are mostly responsible for the health disparities observed within and between countries.^1–6^ The health community has begun to reposition the emphasis of their work from describing the incidence of these disparities to a solution-oriented approach for the whole person—health equity.^7^ Defined as the “attainment of the highest level of health for all people” with intentional efforts to address avoidable inequalities and injustices,^8^ achieving health equity in part requires that the health care field not only acknowledge the overwhelming evidence that health disparities exist, but shift their agenda toward closing the gaps in care and outcomes.

Working toward health care equity (HCE) by eliminating disparities in health care settings represents one component of achieving health equity for all people. Health care-focused organizations like The Joint Commission (TJC) have made suggestions for reducing health disparities. In January 2023, TJC released new HCE accreditation standards (National Patient Safety Goals [NPSG]) for its hospital, ambulatory, and behavioral health and human services accreditation programs, along with a new HCE certification program for hospitals. The standards aim to create a consistent national foundation for the equitable delivery of care. To assist organizations to become compliant with requirements, TJC created HCE resource centers.^9–10^

Research has not yet addressed whether the efforts made by organizations such as TJC are effective in reducing health disparities. The purpose of this study was to assess the current state of the field in terms of its efforts on the journey toward HCE and understand how TJC’s HCE requirements impact the field’s readiness to close gaps in disparate outcomes. The study’s findings will serve as a baseline comparative indicator of the success of components of the accreditation and certification requirements in bringing TJC accredited health care organizations in line with progress toward HCE.

## Methods

### Study Design and Objectives

This was a cross-sectional observational study that consisted of an online questionnaire that assessed four domains related to hospitals' progress toward HCE as it aligns with TJC’s accreditation and certification requirements: 1) leadership, 2) collaboration, 3) collecting and using data, and 4) provision of care.

### Questionnaire Development and Data Collection

The questionnaire consisted of 31 items developed to assess hospitals' implementation of each of TJC’s HCE accreditation standards and certification requirements (Table 1). The HCE accreditation standards are a required component of the accreditation process and have been elevated to NPSG to establish them as a source of major patient harm that all health care settings must address to ensure patient safety. HCE certification requirements are part of a voluntary program covering evaluation of standards compliance and verifying improvement activities. Each NPSG, accreditation standard, and certification requirement includes elements of performance (EPs), which serve as objective measures to assess compliance.

**Table 1.** Summary of TJC Accreditation and Certification HCE Requirements by Questionnaire Topic

Topic area	Summarized HCE NPSG EPs	Summarized HCE certification EPs
Leadership	The hospital designates an individual(s) to lead activities to improve health care equity for the hospital’s patients.	The hospital makes HCE a strategic priority and appoints a leader that understands the needs of the organization’s patient population. The leader provides oversight and coordination of activities to reach its health care equity goals.
Collaboration	The hospital assesses the patient’s health-related social needs and provides information about community resources and support services.	The hospital collaborates with a variety of stakeholders to identify gaps in the delivery of equitable care within and outside of the hospital’s walls.
Collecting and using data	The hospital identifies health care disparities in its patient population by stratifying quality and safety data using the sociodemographic characteristics of the hospital’s patients.	The hospital collects data to understand the sociodemographic characteristics and health-related social needs of the individuals in its community. Data from patients, staff, and leaders should be collected to identify perceptions of bias and to identify organizational opportunities to increase diversity and racial, ethnic, and language concordance.
Provision of care	The hospital develops a written action plan that describes how it will improve health care equity by addressing at least one of the health care disparities identified in its patient population. The hospital acts when it does not achieve or sustain the goal(s) in its action plan to improve health care equity. At least annually, the hospital informs key stakeholders, including leaders, licensed practitioners, and staff, about its progress to improve health care equity.	The hospital supports diversity, equity, and inclusion of staff and leaders and provides the education necessary to support equitable care. The organization communicates effectively with patients, families, and caregivers, accommodates the needs of patients with disabilities, and addresses patients’ health-related social needs.

EPs, elements of performance; HCE, health care equity; NPSG, National Patient Safety Goals; TJC, The Joint Commission.

The study’s purpose and methodology were presented to TJC’s Hospital Advisory Council (HAC), which consists of experts in their field that provide valuable insights to drive TJC’s mission of promoting patient safety and quality. The HAC agreed to pilot test the questionnaire in March 2023. Feedback from 12 pilot test respondents resulted in a slightly refined (e.g., item wording and order) final questionnaire.

TJC accredits 3,820 hospitals. Hospitals that received an accreditation visit in 2022 or who were scheduled for a visit after October 2024 were included in the sampling for this study, leaving 2,098 eligible hospitals. The desired minimum sample size was determined to be 325 respondents, which was calculated based on 5% precision and confidence intervals of 95% after applying a finite population correction factor. With an anticipated response rate of 20%, the study recruitment information was sent to a representative sample of 1,625 hospital’s accreditation contacts for completion by the person(s) with institutional knowledge related to HCE efforts across their organization. The questionnaire was officially launched on April 25, 2023, and closed on June 2, 2023. Respondents were informed that responses would remain anonymous.

### Data Analysis

Descriptive statistics were calculated for each item. Pearson chi-squared tests confirmed significant differences between strata of hospital characteristics. A *p* value of < 0.05 determined statistical significance. For chi-squared tests that included more than two categories of hospital characteristics (i.e., setting, ownership, and bed size), standardized residuals were compared. If the absolute value of the standardized residuals was > 1.96, that characteristic was determined to be driving the significance of the chi-squared result.^11^ Significant differences in responses by hospital characteristics are described in detail in Supplementary Material(Supplementary Data S1–S4). Percentages in text and tables may not add to 100% due to respondent’s ability to select multiple responses and skip items.

## Results

### Respondent Characteristics

Three hundred forty hospitals responded to the questionnaire (20.9% response rate). Responding hospitals represented a range of types, most being community hospitals (140, 41.2%). In total, 224 (65.9%) hospitals were part of a health care system. Hospitals' ownership status was mostly not-for-profit (188, 55.3%). Many hospitals (191, 56.2%) were mid-size to large (i.e., 100–499 beds; Table 2). Individual respondents were mostly from top management positions, such as Chief Quality Officer, Chief Compliance Officer, Chief Nursing Officer, Chief Operations Officer, and Chief Executive Officer. About 24 (7.1%) respondents indicated holding a leadership role specific to diversity, equity, or inclusion (e.g., Chief Equity Officer).

**Table 2.** Self-Reported Respondent Characteristics

Hospital characteristic	*N* (%)
Hospital type	
Community	140 (41.2%)
Academic Medical Center/Teaching	53 (15.6%)
Behavioral Health/Psychiatric	48 (14.1%)
Rural	34 (10.0%)
Specialty	17 (5.0%)
Long-term acute	11 (3.2%)
Federally Qualified Health Center	7 (2.1%)
Children’s	5 (1.5%)
System	
Yes	224 (65.9%)
No	114 (33.5%)
Ownership	
Not-for-profit	188 (55.3%)
For-profit	80 (23.5%)
Government	69 (20.3%)
Bed capacity	
<100	103 (30.2%)
100–499	191 (56.2%
>500	42 (12.5%)

Compared with the sample from which they were drawn, academic medical centers/teaching hospitals were overrepresented in the response group (6.9% of sample, *p* < 0.05), and community hospitals were underrepresented (52.5% of sample, *p* < 0.05). System-based hospitals were underrepresented in the response group (80.3% of sample, *p* < 0.001). Not-for-profit hospitals were overrepresented in the response group (49.4% of sample, *p* < 0.05).

### Leadership

Most hospitals (248, 73.4%) reported having a designated leader with HCE as a defined responsibility. Some hospitals (71, 21.1%) have a standalone HCE strategic plan, while about half (169, 50.1%) have components of HCE plans incorporated into other strategic plans (e.g., human resources plan). Some hospitals have an HCE plan in development (78, 23.1%) with timelines for completion described as stretching from mid-2023 to early 2024, whereas a small number do not have a plan at all (19, 5.6%).

### Collaboration

About half of the hospitals (173, 51.3%) reported intentionally seeking input from patients and families in a structured way when developing and updating their HCE procedures. Many of these hospitals (96, 55.5%) established a permanent patient and family advisory group with diverse membership to collect information about the experience of equitable care. Some (76, 43.9%) used alternative strategies to engage patients and families (e.g., questionnaires, individual interviews, and patient-included rounds). Some (57, 32.9%) conducted focus groups with patients and families or held meetings in the community to learn about their impressions and experiences (56, 32.4%).

Many hospitals (197, 58.8%) reported formally seeking input from established community organizations when developing and updating their HCE procedures. Most of these hospitals (184, 92.5%) established ongoing collaborations with community organizations. When asked to describe the relationship between their hospital and service organizations in the community, many reported either having a list of organizations to which they refer patients without a follow-up process (81, 44.0%) or referring patients to organizations with a reciprocal process for sharing patient progress (77, 41.8%). Fewer hospitals (21, 11.4%) have a closed-loop referral process, whereby technology documents that a patient is referred, resources are secured, and needs are addressed.

### Collecting and Using Data

Most hospitals (320, 95.0%) require staff to ask about and collect patients’ self-reported race/ethnicity and provide staff with training about how to sensitively collect the information (261, 77.7%). Many hospitals (196, 58.7%) do not audit the proportion of patients classified as having “missing” or “unknown” race/ethnicity data to improve the accuracy and completeness of the data collection process.

Many hospitals (236, 70.4%) reported analyzing health care quality and safety measures to determine whether there are disparities in care. Slightly over half of hospitals (187, 55.8%) analyze patient experience of care measures for disparities. Hospitals used race (221, 93.6% for quality and safety; 163, 87.2% for experience of care) most frequently to stratify data (Table 3). Hospitals reported prioritizing readmissions (163, 69.1%), diabetes (117, 49.6%), maternal morbidity (108, 45.8%), cardiovascular disease (95, 40.3%), hospital-acquired conditions (87, 36.9%), hypertension (72, 30.5%), fall rates (69, 29.2%), immunization rates (66, 28.0%), cancer screening (64, 27.1%), restraint use (53, 22.5%), asthma (28, 11.9%), and kidney transplantation rates (11, 4.7%) when routinely analyzing data to assess or address inequities.

**Table 3.** Patient Characteristics by Which Hospitals Reported Analyzing Data for Disparities

Patient characteristics	By quality and safety measures *N* (% of 236)	By patient experience of care *N* (% of 187)
Race	221 (93.6%)	163 (87.2%)
Ethnicity	213 (90.3%)	146 (78.1%)
Sex assigned at birth (i.e., male/female)	189 (80.1%)	124 (66.3%)
Language (i.e., preferred language or English-speaking status)	177 (75.0%)	124 (66.3%)
Social determinants of health	149 (63.1%)	85 (45.5%)
Insurance coverage	144 (61.0%)	59 (31.6%)
Income/poverty level (e.g., community-level data)	92 (39.0%)	51 (27.3%)
Other (please specify)	27 (11.4%)	22 (11.8%)

Most hospitals (216, 64.9%) use data to examine the adequacy of their interpreter services. Many review all aspects of their language access services, such as whether an interpreter is offered (147, 68.1%), provided (159, 73.6%), and services cover the necessary range of languages for the population (173, 80.1%). Over half of the hospitals (190, 56.5%) reported having a process to assess whether staff have adequate language proficiency when communicating with patients in a language other than English without an interpreter.

Most hospitals (215, 64.4%) reported collecting data about incidents and perceptions of discrimination experienced by staff members and analyze it by race (187, 87.0%), ethnicity (176, 81.9%), language (132, 61.4%), and gender (170, 79.1%).

### Provision of Care

Most hospitals (210, 62.1%) routinely screen as many patients as possible for health-related social needs (HRSN), while a lesser proportion (99, 29.3%) only screen if there is an indication of an issue, or do not screen at all (29, 8.6%). Of those hospitals that screen for HRSN in any capacity (309, 91.4%), most reported screening for each need listed in the questionnaire (Table 4).

**Table 4.** Patients’ Health-Related Social Needs for Which Hospitals Screen

HRSN domain	*N* (% of 309)
Housing instability	276 (89.3%)
Transportation needs	270 (87.4%)
Food insecurity	264 (85.4%)
Interpersonal safety	245 (79.3%)
Difficulty paying for prescriptions	222 (71.8%)
Difficulty paying for medical bills	177 (57.3%)
Utility difficulties	155 (50.2%)
Other (please specify)	28 (9.1%)

HRSN, health-related social needs.

Half of the hospitals (168, 50.6%) compare the diversity of their employees with that of their patient population to assess concordance in characteristics, such as race, ethnicity, and languages spoken. Over half (185, 56.2%) set a goal to increase recruitment of employees with diverse backgrounds to achieve greater concordance.

### Variations by Hospital Characteristics

Hospital characteristics were significantly associated with implementation of the HCE components assessed in this study.

#### Leadership

When stratifying by hospital characteristics, behavioral health/psychiatric hospitals and small hospitals were less likely to have a designated HCE leader. Not-for-profit hospitals were more likely to have a designated HCE leader and a standalone strategic plan. Free-standing hospitals were less likely to have a strategic plan (S1).

#### Collaboration

Behavioral health/psychiatric and long-term acute care hospitals were less likely to seek formal input from the community and establish ongoing collaborations with community organizations. Not-for-profit hospitals and hospitals that are part of a health care system were more likely to seek community input and establish community collaborations (S2).

#### Collecting and Using Data

Free-standing and government hospitals were less likely to provide sensitive information collection training, whereas not-for-profit and large hospitals were more likely to provide this training. Behavioral health/psychiatric, free-standing, and small hospitals were less likely to analyze quality and safety measure data for disparities in care, and behavioral health/psychiatric hospitals were less likely to analyze experience of care data. Government-owned hospitals were less likely to examine the adequacy of their interpreter services (S3).

#### Provision of Care

Academic medical centers/teaching hospitals and large hospitals were more likely to assess the concordance of demographic characteristics between patients and employees. Hospitals that are part of a health care system were more likely to set a recruitment goal to achieve concordance (S4).

## Discussion

This study assessed the current state of the field’s efforts toward HCE as it aligns with four components of TJC’s accreditation and certification requirements: 1) leadership, 2) collaboration, 3) collecting and using data, and 4) provision of care. In general, many of the hospitals surveyed have made advances in the topics covered in this study, but some have only done so to a limited degree.

Although most hospitals have designated a leader of HCE efforts, over a quarter have not yet done so. Likewise, most hospitals have strategic plans (or plans in development) to address HCE, but only a fifth have a standalone strategic plan guiding their work. Those in the process of developing their HCE strategic plan anticipate completion timelines that fall within 6 months to 1 year of the release of TJC’s HCE standards. It may be that release of the standards prompted the creation of previously nonexistent HCE strategic plans. Small hospitals are comparatively less likely to have HCE leadership appointments and plans to address HCE. Although TJC requirements do not prescribe the percent effort of HCE leaders, small hospitals may lack desired resources to cover the cost of leadership recruitment, promotion, and/or development programs.^12^

Only half of hospitals engaged with patients, families, and community social service organizations to develop HCE procedures. Hospitals that are collaborating often do so without a follow-up process or with an informal information sharing process. It is noteworthy that very few hospitals are achieving the gold standard,^13^ closed-loop referral—a technology-enabled process which ensures that care teams connect patients with resources to meet their needs, patients use those resources, and care teams receive feedback about outcomes and next steps. Closed-loop referral systems often use advanced technological infrastructures to which some hospitals might not have access. In addition, they often require community-based organizational availability, which could be lacking in certain geographical locations, and specific incentives or payment structures to foster buy-in. Hospitals not pursuing collaboration may have differences in patient services or governance. For example, long-term acute care hospitals serve patients with such complex medical needs^14^ that collaboration with a local food bank to address food insecurity may not be an immediate, or even long-term, strategy to care for the person. Other hospitals, like those that are government-owned, may lack the autonomy to collaborate externally^15^ due to priorities set by overarching laws, regulations, and policies rather than the local community.

Almost all hospitals surveyed collect race and ethnicity data from their patients in a sensitive manner. Research has shown that data on patients' race and ethnicity are frequently missing or incomplete.^16^ Hospitals in this study did not report auditing practices for race and ethnicity data, which likely means data quality issues remain. High rates of missing data can impact data analyses and obscure inequities that may be present had complete and accurate data been used. Hospitals in this study report examining a variety of medical outcomes for gaps in care. The outcomes examined most frequently may be those for which interventions are abundant in the literature.^17–19^ There is still a large portion of hospitals that are not yet looking at their data for disparities in quality, safety, experience of care, medical outcomes, or even the adequacy of interpreter services. These hospitals may not yet have the technological infrastructure necessary to look closely at these data^20^ or are unaware of the business value of adopting and implementing the infrastructure.^21^ TJC’s HCE standards could serve as a catalyst to building or improving upon these processes.

As the health community moves from collecting HRSN data that describe disparities to practicing solution-based health care equitably,^7^ hospitals that are not yet learning about the needs of their patient populations may fall behind in the equitable provision of care. Some hospitals are doing at least some screening for HRSN, which often represents the first step of an individual-level acute intervention. For example, many are inquiring about housing instability, food insecurity, transportation needs, interpersonal safety, and ability to pay for prescriptions, while fewer are assessing for difficulties paying for utilities and medical bills. Of note is the moderate portion of hospitals not screening patients at all. Scientific evidence and awareness about the impact of social determinants of health (SDOH) on population health and well-being are mounting,^2–6^ and it is crucial that the health system respond accordingly by building upon the collection of HRSN targeting the immediate social needs of patients to inform long-term changes that target community conditions (i.e., SDOH). Large and academic/teaching hospitals are more likely to assess the current state of employee diversity, which aligns with their general vision that it is imperative for the medical community to contribute to the design and implementation of solutions that directly address racism.^22^

Hospitals with certain characteristics, like those that are not-for-profit or part of a health care system, are more consistently implementing many HCE components. Because the mission of not-for-profit hospitals is often to serve the broader community, with a focus on charitable actions and elevated levels of uncompensated care,^12^ they may approach equitable care as a value that is inherently woven into the fabric of their institution. Hospital systems often serve as vehicles for diffusing practices throughout a collection of individual hospitals.^23^ Sharing knowledge, coordination of services, reduced duplication, and efficiency^24^ may allow systems to access more financial resources^12^ that can be devoted to HCE efforts.

Behavioral health/psychiatric hospitals appear to be lagging on addressing HCE. Some evidence suggests that psychiatric hospitals use electronic health records (EHR) at a lower rate compared with general medicine and surgical practices^25^ because they are often unable to invest in the hardware, software, and training necessary for EHR adoption.^26^ Behavioral health/psychiatric hospitals may struggle to meet HCE standards because they are not accessing the EHR, a core piece of technology that facilitates much of this work. Alternatively, it may be that behavioral health/psychiatric hospitals have lower operating margins that prevent them from not only improving information technology systems but also reaching out beyond their institution’s walls to address the social needs of their patient population.

### Limitations

The response rate for this study was low. There was an attempt to mitigate participant response fatigue by including binary response questions in the survey, but the questionnaire contained over 30 questions and response rates to later questions decreased compared with questions presented earlier. Some hospital types were over or underrepresented in the response data compared with the overall sample. Given that certain hospital characteristics were strongly associated with implementation of certain HCE activities, and some of these hospital types were overrepresented among responders, it is possible that results are an overestimate of progress across the field in general. Importantly, responses are likely to suffer from some degree of favorability bias. While variation in implementation of HCE actions existed among responders, it seems likely that responders may have been further along in their HCE journey than non-responders. Because questions were related to accreditation and certification requirements, responders may have been more likely to report compliance (whereas nonresponders may have opted out to avoid any appearance of noncompliance with standards). Results, therefore, are likely to overestimate progress. Despite these limitations, the study identified significant gaps in the implementation of core HCE activities.

### Future Directions

The state of the field should be reassessed after hospitals are allotted time to become compliant with TJC HCE standards. Linking the findings to community and health care worker characteristics can assess unique differences between hospitals' HCE progress. The recent media implications that some health care organizations are stepping back from a HCE focus are important. Understanding both internal (e.g., staff) and external (e.g., legislative) dissension could shed light on the future of health equity.

## Health Equity Implications

Most hospitals have taken initial actions to address HCE, assigning leaders, and setting strategic priorities. But considerable progress is still needed to address gaps in data collection, the use of data, and the provision of care. Not-for profit hospitals and those that are part of a system have made greater progress toward achieving compliance with the HCE requirements, whereas behavioral health/psychiatric hospitals have some work to do to make headway in this area. HCE is necessary to progress toward health equity, but it is not the whole answer. Institutions must work together to achieve health equity for all people and address SDOH for entire communities in addition to individual patient needs.

## Abbreviations Used

EHR: Electronic health record
EPs: Elements of performance
HAC: Hospital Advisory Council
HCE: Health care equity
HRSN: Health related social needs
NPSG: National Patient Safety Goals
SDOH: Social determinants of health
TJC: The Joint Commission
