# Supplementary material for: The Journey Toward Health Care Equity: Accredited Hospitals’ Alignment with Joint Commission Health Care Equity Standards Preimplementation
Source: Health Equity. 2024 Oct 23;8(1):738–45. doi: 10.1089/heq.2024.0036 (PMC11512081; doi:10.1089/heq.2024.0036)
Supplement: Supplemental Data S1 [file heq.2024.0036_supplemental_data.docx]

# **S1.**

Leadership Results Stratified by Hospital Characteristics

**Table S1.1.** Hospitals that have a designated HCE leader by characteristic.

| Designated HCE Leader | % | P-Value |
| --- | --- | --- |
| Hospital Type |  |  |
| Behavioral Health/Psychiatric | 58.3% | *p*<.05 |
| Academic/Teaching | 86.8% |  |
| Children’s | 80.0% |  |
| Community | 79.1% |  |
| Specialty | 75.0% |  |
| Bed Capacity | | |
| <50 | 65.3% | *p*<.05 |
| 50-99 | 57.7% |  |
| 100-299 | 75.8% |  |
| 300-500 | 76.3% |  |
| >500 | 88.1% |  |
| Ownership | | |
| Not-for-profit | 80.7% | *p*<.01 |
| For-profit | 62.0% |  |
| Government | 65.2% |  |

**Table S1.2** Hospitals that have a standalone HCE strategic plan by characteristic.

| Standalone HCE Strategic Plan | % | P-Value |
| --- | --- | --- |
| Ownership |  |  |
| Not-for-profit | 27.8% | *p*<.01 |
| For-profit | 12.5% |  |
| Government | 11.6% |  |
| System Member |  |  |
| Yes | 25.0% | *p*<.001 |
| No | 12.6% |  |

# **S2.**

Collaboration Results Stratified by Hospital Characteristics

**Table S2.1.** Hospitals that seek formal input from the community by characteristic.

| Seek Community Input | % | P-Value |
| --- | --- | --- |
| Hospital Type |  |  |
| Behavioral Health/Psychiatric | 31.3% | *p*<.001 |
| Academic/Teaching | 73.1% |  |
| Children’s | 80.0% |  |
| Community | 64.7% |  |
| Specialty | 57.1% |  |
| Ownership | | |
| Not-for-profit | 71.0% | *p*<.001 |
| For-profit | 50.6% |  |
| Government | 36.2% |  |
| System Member | | |
| Yes | 64.3% | *p*<.01 |
| No | 47.7% |  |

**Table S2.2.** Hospitals that have established ongoing collaborations with community organizations by characteristic.

| Ongoing Collaborations | % | P-Value |
| --- | --- | --- |
| Hospital Type |  |  |
| Behavioral Health/Psychiatric | 66.7% | *p*<.001 |
| Long term acute | 50.0% |  |
| Academic/Teaching | 97.4% |  |
| Children’s | 100.0% |  |
| Community | 95.6% |  |
| Specialty | 91.7% |  |
| Ownership | | |
| Not-for-profit | 98.5% | *p*<.001 |
| For-profit | 78.0% |  |
| Government | 84.0% |  |
| System Member | | |
| Yes | 95.1% | *p*<.05 |
| No | 85.2% |  |

# **S3.**

Collecting & Using Data Results Stratified by Hospital Characteristics

**Table S3.1.** Hospitals that provide training to collect sensitive information by characteristic.

| Sensitive Information Training | % | P-Value |
| --- | --- | --- |
| Bed Capacity |  |  |
| <50 | 70.0% | *p*<.05 |
| 50-99 | 73.1% |  |
| 100-299 | 80.9% |  |
| 300-50 | 69.5% |  |
| >500 | 92.9% |  |
| Ownership | | |
| Not-for-profit | 84.9% | *p*<.001 |
| For-profit | 76.3% |  |
| Government | 59.4% |  |
| System Member | | |
| Yes | 83.9% | *p*<.001 |
| No | 65.8% |  |

**Table S3.2.** Hospitals that analyze quality and safety measure data for disparities in care by characteristic.

| Analyze Quality and Safety Data | % | P-Value |
| --- | --- | --- |
| Hospital Type |  |  |
| Behavioral Health/Psychiatric | 52.1% | *p*<.01 |
| Academic/Teaching | 81.1% |  |
| Children’s | 100.0% |  |
| Community | 73.6% |  |
| Specialty | 81.3% |  |
| Bed Capacity |  |  |
| <50 | 67.3% | *p*<.05 |
| 50-99 | 55.8% |  |
| 100-299 | 72.0% |  |
| 300-50 | 70.7% |  |
| >500 | 88.1% |  |
| System Member | | |
| Yes | 77.9% | *p*<.001 |
| No | 55.9% |  |

**Table S3.3.** Hospitals that analyze experience of care data for disparities by characteristic.

| Analyze Experience of Care Data | % | P-Value |
| --- | --- | --- |
| Hospital Type |  |  |
| Behavioral Health/Psychiatric | 31.9% | *p*<.05 |
| Academic/Teaching | 62.3% |  |
| Children’s | 80.0% |  |
| Community | 60.0% |  |
| Specialty | 50.0% |  |

**Table S3.4.** Hospitals that use data to examine the adequacy of their interpreter services.

| Examine Interpreter Services | % | P-Value |
| --- | --- | --- |
| Ownership |  |  |
| Not-for-profit | 67.6% | *p*<.01 |
| For-profit | 72.5% |  |
| Government | 48.5% |  |

# **S4.**

Provision of Care Results Stratified by Hospital Characteristics

**Table S4.1.** Hospitals that assess the concordance of their employee and patient demographics by characteristic.

| Analyze Quality and Safety Data | % | P-Value |
| --- | --- | --- |
| Hospital Type |  |  |
| Behavioral Health/Psychiatric | 33.3% | *p*<.05 |
| Academic/Teaching | 73.1% |  |
| Community | 49.6% |  |
| Specialty | 52.9% |  |
| Bed Capacity |  |  |
| <50 | 38.0% | *p*<.01 |
| 50-99 | 44.2% |  |
| 100-299 | 49.6% |  |
| 300-50 | 52.6% |  |
| >500 | 75.6% |  |
| System Member | | |
| Yes | 77.9% | *p*<.001 |
| No | 55.9% |  |

**Table S4.2.** Hospitals that have set a goal to increase the number of employees with diverse backgrounds to achieve greater concordance with their patient population by characteristic.

| Analyze Quality and Safety Data | % | P-Value |
| --- | --- | --- |
| System Member | | |
| Yes | 61.0% | *p*<.05 |
| No | 46.4% |  |
